# Photoacoustic imaging reveals hidden underdrawings in paintings

**DOI:** 10.1038/s41598-017-00873-7

**Published:** 2017-04-07

**Authors:** George J. Tserevelakis, Ilianna Vrouvaki, Panagiotis Siozos, Krystallia Melessanaki, Kostas Hatzigiannakis, Costas Fotakis, Giannis Zacharakis

**Affiliations:** 1 Institute of Electronic Structure and Laser, Foundation for Research and Technology Hellas, Heraklion, Crete Greece; 2 Department of Chemistry, University of Crete, Heraklion, Crete Greece; 3 Department of Physics, University of Crete, Heraklion, Crete Greece

## Abstract

A novel, non-invasive, imaging methodology, based on the photoacoustic effect, is introduced in the context of artwork diagnostics with emphasis on the uncovering of hidden features such as underdrawings or original sketch lines in paintings. Photoacoustic microscopy, a rapidly growing imaging method widely employed in biomedical research, exploits the ultrasonic acoustic waves, generated by light from a pulsed or intensity modulated source interacting with a medium, to map the spatial distribution of absorbing components. Having over three orders of magnitude higher transmission through strongly scattering media, compared to light in the visible and near infrared, the photoacoustic signal offers substantially improved detection sensitivity and achieves excellent optical absorption contrast at high spatial resolution. Photoacoustic images, collected from miniature oil paintings on canvas, illuminated with a nanosecond pulsed Nd:YAG laser at 1064 nm on their reverse side, reveal clearly the presence of pencil sketch lines coated over by several paint layers, exceeding 0.5 mm in thickness. By adjusting the detection bandwidth of the optically induced ultrasonic waves, photoacoustic imaging can be used for looking into a broad variety of artefacts having diverse optical properties and geometrical profiles, such as manuscripts, glass objects, plastic modern art or even stone sculpture.

## Introduction

Visible optical imaging, including optical microscopy, often enhanced with multi-spectral resolution, represents a powerful approach in the investigation of various types of artworks, particularly those bearing color such as easel or wall paintings and polychromies. Spectrally-resolved imaging enables mapping of different paints across the surface of a painting and in many cases the recovered spectral information leads to pigment identification.

However, paintings typically consist of several successive strata of paint, which are optically opaque, primarily because of strong scattering arising from the pigment particles. As a result, visible optical imaging is rather limited to a superficial analysis and thus features, which have been painted over, and can be significant for evaluating the previous history of a painting or even the making of it, may escape detection. This problem is directly related to the nature of light transport through opaque media, which is governed by scattering and diffusion that scramble any spatial information the same way fog blurs our vision. Therefore, there is an evident need for the development of novel, high precision, non-destructive imaging tools that will surpass the limitations imposed by light scattering and hence, extend the depth from which accurate information can be extracted.

In this context, a challenge art conservation scientists often face relates to the detection and mapping of underdrawings in paintings. Underdrawings typically represent the original rough sketch used by the artist to portray the theme of the painting and guide the development of the artwork. Such kind of preparatory sketching is generally applied directly on the prepared canvas using charcoal, graphite or carbon black paint. Unavoidably, the covering of the preliminary drawing with several layers of paint, obstructs locating and retrieving the information related to the initial part of the artistic process, namely the original sketch, and furthermore the degree to which the artist followed faithfully this sketch or introduced subsequent alterations in the artwork (pentimento). Underdrawings or underpaintings have been of exceptional interest to scholars for long time, because they contain information about the working practice of a particular artist or workshop. More specifically, the non-destructive detection of such hidden features has the potential to shed light to the social, historical, geographical or even psychological framework of the artistic creation, providing thus invaluable knowledge not only to art historians, but also to cultural heritage scientists who are interested in the determination of artworks authenticity. It is worth mentioning that there are numerous bibliographical references stating case-studies of underdrawings detection in historical paintings, proving thus, the huge interest of the artistic community for such applications. Indicatively, we report the attempts that have been made to detect hidden features in “Virgin of the rocks” by Leonardo Davinci^1^, “An Old Man in Military Costume” by Rembrandt van Rijn^2^, “L’ultimo bacio dato a Giulietta da Romeo” by Francesco Hayez^3^, “Patch of Grass” by Vincent van Gogh^4^, “Portrait of General John P. McCown” by Lloyd Branson^5^, Several academic nude paintings by José Veloso Salgado^6^, “Portrait of an Old Man” by Rembrandt van Rijn^7^, “Portrait of an old musician with flute” by Thomas Faed^8^, and “The blue room” by Pablo Picasso^9^.

The state of the art and the technique most exploited to date for detecting and mapping underdrawings in paintings, is Near Infrared (NIR) Reflectance Imaging^10–12^, typically employing radiation in the wavelength range of 0.8–3 μm. The principle behind this methodology relies on the reduced absorption and scattering of pigment materials in the NIR, which permits light to penetrate deeper within the painting and provide information about features situated immediately underneath the top paint layers, albeit within a range no deeper than 200–300 μm. In particular, dark materials or pigments, used for sketch lines and underdrawings, absorb the incident NIR radiation, more strongly than other pigments, which exhibit relatively high transparency in this spectral range due to the absence of strong electronic transitions. In such instances, the reflected intensity distribution in the NIR image of the painting is drastically affected by the presence of the highly absorbing underdrawing material, which permits mapping the underlying sketch by use of a NIR sensitive CCD camera.

Despite its broad application, NIR imaging is not always capable of providing strong contrast of the initial sketch, especially when the overlying pigments are not adequately transparent in the NIR. An additional drawback of this modality is that the imaging efficiency is very much dependent on the paint layer thickness and therefore, regions of the artwork covered by relatively thick paint layers cannot be successfully resolved due to the intense scattering of the incident radiation limiting optical penetration.

Other state of the art technologies for mapping underdrawings include Optical Coherence Tomography (OCT)^13, 14^, THz radiation imaging^15–17^ or X-ray based imaging^18, 19^. OCT is an optical coherence diagnostic technique offering high spatial resolution in 3-D but is still limited with respect to depth when highly scattering media, such as paints, are probed. As a result, OCT can provide information exclusively in special cases concerning very thin paint layers with a high degree of transparency in the NIR. On the other hand, THz radiation imaging offers higher penetration capabilities within optically opaque media and has been demonstrated to be a promising tool for underdrawing visualization. Nevertheless, such an approach requires a rather complex and expensive experimental apparatus, incorporating fs lasers, lock-in amplifiers, THz spectrometers and other pieces of high cost equipment. Furthermore, both the contrast and the spatial resolution of the recorded images are relatively low, not allowing for the precise discrimination of several details in the underlying hidden sketch. Finally, X-ray radiography has also been extensively used as a diagnostic tool providing high resolution images of non-visible features hidden below the paint layers. The main drawback of X-rays however, is their rather low transmittance through pigments, containing high atomic number elements, such as white lead, which was in fact the only white paint broadly available to artists until the early twentieth century. Hence, the superficial X-ray absorption prevents the delineation of the underdrawing pattern in many historical paintings of interest. Recently, X-ray fluorescence (XRF)^20, 21^ elemental mapping of whole paintings has been achieved and provides rich information on the distribution of pigments across the painting surface, however sketches and underdrawings made of carbon-based materials are hardly visible by XRF and this holds for X-ray radiography as well.

These methods have also been widely employed in biomedical imaging research and practice^22^ due to the very similar interactions of biological media with several forms of electromagnetic radiation, which provides the driving idea of adapting a wealth of novel imaging technologies that offer insight to opaque media.

In a predominant position, in recent years, among imaging technologies we find photoacoustic imaging, a novel methodology developed in the context of contemporary biomedical research studies and enabling *in vivo* high resolution imaging of biological substrates, ranging from cells and tissues to organs and small animals. Noteworthy are preclinical applications of photoacoustic imaging in the high resolution imaging of cancer tumors^23–25^, the study of metabolic disorders by mapping oxygen saturation of hemoglobin^26, 27^, or the investigation of drug release processes^28–31^.

According to the photoacoustic effect, when light of time-variable intensity (e.g. a laser pulse) is absorbed by a material, the thermoelastic expansion of the medium will give rise to a rapid pressure change, which propagates in the form of acoustic waves into the surrounding environment. For imaging applications, the frequency range of the detected photoacoustic signals is in the ultrasonic regime, usually varying from a few up to several tens of MHz. The main advantage of this approach lies on the ability of the technique to provide optical absorption contrast in turbid media, while retaining high spatiotemporal resolution. The trade-off between imaging depth and resolving spatial detail can be generally adjusted according to the detection bandwidth of the optically induced ultrasonic waves, permitting thus the investigation of a vast variety of specimens with diverse optical and geometrical properties.

In this work, we demonstrate how photoacoustic imaging can break the barriers of biomedicine, and find innovative applications in artwork diagnostics. By employing an acoustic resolution photoacoustic microscopy (AR-PAM) approach, we establish a novel, non-destructive, and universal methodology for the precise delineation of underdrawings in paintings. It is shown that the intrinsically hybrid nature of photoacoustic imaging can overcome several major restrictions of other imaging techniques, offering improved detection sensitivity and contrast at high spatial resolution.

## Results

### Photoacoustic imaging apparatus

**Figure 1:**
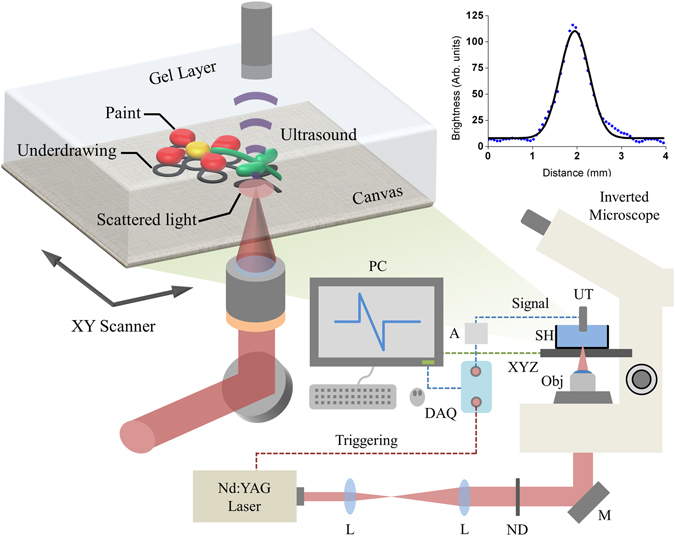
Photoacoustic imaging apparatus and sample configuration. Abbreviations: L, Lens; ND, Neutral density filters; M, Mirror; Obj, Objective lens; SH, Sample holder; XYZ, 3D translational stage; UT, Ultrasonic transducer; A, Amplifier; DAQ, Data acquisition card; PC, Recording computer. Inset shows the lateral profile of the irradiating beam on the canvas surface (dots: experimental points, solid line: fit to a Gaussian; R^2^ = 0.988).

The photoacoustic imaging apparatus employed in the present study (Fig. 1, Methods section) has been developed by modifying a conventional inverted optical microscope. Nanosecond pulses at λ = 1064 nm, emitted by a Q-switched Nd:YAG laser, focused by a low numerical aperture objective lens, are incident on the reverse side of the painting, namely on the canvas support. Strong light scattering generates a broad illumination spot, inducing the generation of photoacoustic waves in the highly absorbing regions of the underdrawing pattern. Since the NIR transparent overlying paint layers have a virtually zero contribution in the image formation, the signals reveal exclusively the hidden sketch at a high contrast level. The photoacoustic signal is detected by a spherically focused ultrasonic transducer immersed in a carboxymethyl cellulose (CMC) gel layer (Methods section) applied on the front surface of the painting and serving as an immersion medium for the effective ultrasound wave propagation from the substrate to the detector. The detection bandwidth of the generated photoacoustic waves is centered at 20 MHz, providing thus a benign trade-off between signal to noise ratio and spatial resolution. The photoacoustic image is generated by raster scanning of the painting using a motorized XY stage to achieve a point by point data acquisition over the region of interest.

### Local temperanture rise estimation

Assuming that heat dissipation is negligible during the laser pulse, the local temperature rise, ΔT, in the irradiated region can be approximated by the expression^32^

1 $$\begin{array}{ccc}{\rm{\Delta }}T & = & \frac{{\eta }_{th}{A}_{e}}{\rho {C}_{V}}\end{array}$$

where *η*_*th*_ stands for the percentage of the pulse energy converted into heat, *A*_*e*_ is the specific optical absorption (J/m^3^), *ρ* is the medium mass density and *C*_*V*_ denotes the respective specific heat capacity at a constant volume. The specific optical absorption can be adequately approximated as the product of the energy fluence, *F*, and the absorption coefficient of the medium *μ*_*α*_.

To estimate accurately the area irradiated by the laser in the plane of the underdrawing layer, a CCD camera is used to image the forward scattered intensity distribution emerging from a piece of canvas positioned in place of the painting and irradiated on its back side. The intensity profile of the resulting laser spot is nearly Gaussian (Fig. 1, inset) with its 1/e^2^ width equal to 1.31 mm. By considering this value as the effective diameter of the energy deposition region and by taking into account that the pulse energy has been measured to be around 1.14 μJ (Methods section), a laser fluence value on the order of 0.085 mJ/cm^2^ is calculated as incident on the underdrawing layer assuming 100% transmission of the canvas, which is not true, hence it sets an upper limit of the laser fluence. Since the absorption coefficient *μ*_*α*_ of graphite, which is the main absorbing material in the examined paintings for the Nd:YAG line^33^, has a value of 2.4 × 10^5^ cm^−1^, *A*_*e*_ ≈ 20 J/cm^3^. For typical values of graphite density^34^ and specific heat capacity^35^ at room temperature (*ρ* = 2.25 g/cm^3^ and *C*_*V*_ = 0.7069 JK^−1^g^−1^ respectively), Eq. 1 yields a maximum (*η*_*th*_ = 1) local temperature rise of ΔΤ ≈ 12.6 Κ per laser pulse incident on the sample.

### Qualitative evaluation of photoacoustic imaging performance

**Figure 2:**
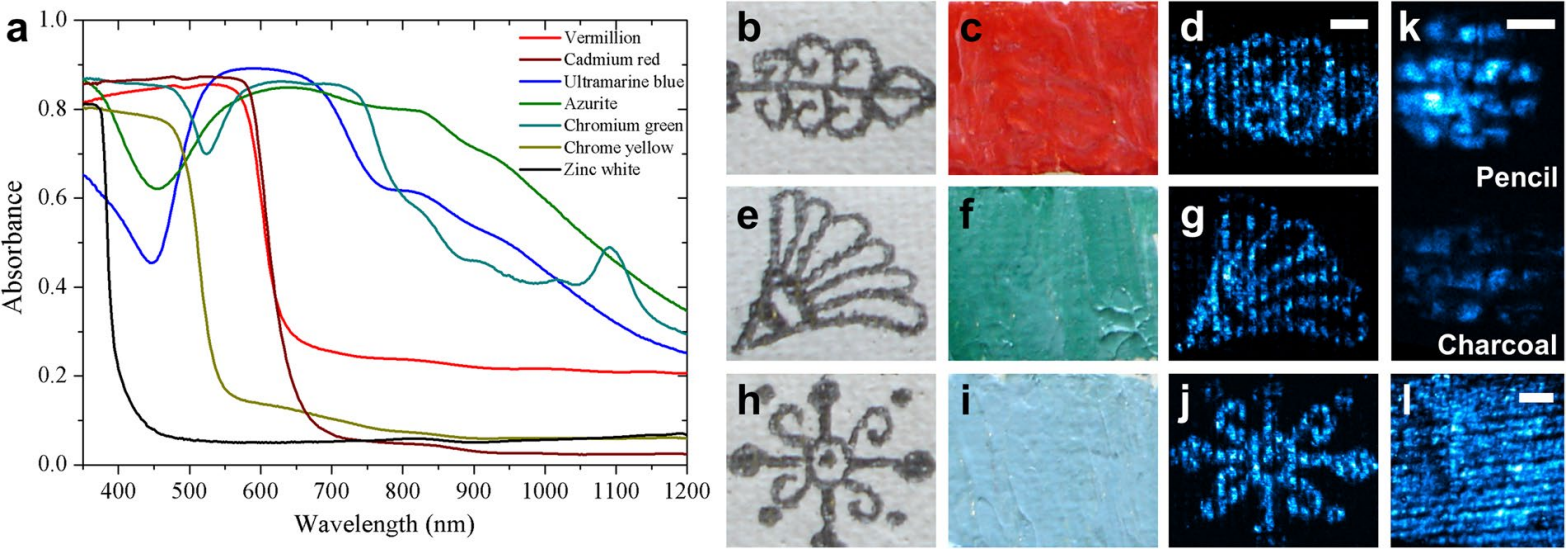
Photoacoustic imaging of various test paint samples. (**a**) Vis-NIR absorption spectra collected in diffuse reflectance mode of test pigments. (**b**) Brightfield view of a pencil sketch on canvas. (**c**) Brightfield view of the previous sample overpainted with a red paint, vermillion. (**d**) Recovered pattern of the initial underlying sketch through photoacoustic imaging. Scalebar: 2 mm. (**e**–**g** and **h**–**j**) Images for chromium green and ultramarine blue paints and corresponding underdrawings respectively. (**k**) A direct photoacoustic signal comparison between pencil and charcoal sketches coated with a zinc white paint layer. Scalebar: 1 mm. (**l**) Background photoacoustic image of a homogeneously pencil-darkened canvas sample. Scalebar: 2 mm.

A series of mock-up oil paint samples of different colors were prepared on small pieces of prepared canvas bearing pencil drawings. These samples were used to test the photoacoustic imaging system and evaluate its capacity with emphasis on underdrawing detection and imaging. Several synthetic inorganic pigments were selected reflecting a palette extensively employed by artists during the 19^th^ and early 20^th^ century, and representative of many of the paintings generated during that period^36, 37^. The absorption spectra collected from these pigments in powder form in the spectral region of 350–1200 nm are shown Fig. 2a. Evidently the observed absorption features correlate with the color of each pigment but it is also noted that these pigments show significantly different absorption at 1064 nm, the emission line of the Nd:YAG laser. For example, the relative absorbance ranges from as low as 3% for cadmium red and up to 50% for azurite, which permits the evaluation of the photoacoustic modality performance under the varying imaging conditions that arise from the different absorbance of the paint covering the pencil underdrawing. In this direction, the qualitative capability of underdrawings delineation was initially tested by measuring three paint samples corresponding to the primary colors, red, green and blue. More specifically, Fig. 2b displays the brightfield image of a pencil sketch on a canvas sample, which was subsequently coated over with a layer of red paint, vermillion (Fig. 2c). The corresponding maximum amplitude projection photoacoustic image of the red paint sample, shown in Fig. 2d, reveals clearly and with adequate resolution the underlying drawing. Likewise similar sketches on canvas covered by chromium green paint (Fig. 2e–g) or ultramarine blue (Fig. 2h–j) are clearly imaged by the photoacoustic system. It is noted that the regions appearing in the images as having reduced contrast correspond to the canvas fiber grid, where scattering of the incident laser irradiation increases, and locally hinders the respective photoacoustic excitation of the underdrawing.

Furthermore, to demonstrate the effectiveness of detecting underdrawings made of different materials, we performed a direct photoacoustic imaging comparison between two simple underdrawing patterns, sketched on the same canvas using pencil (graphite) and a charcoal-based pigment. The sketches were subsequently covered by a paint layer of zinc white, presenting minimal absorption in the excitation wavelength. The results shown in Fig. 2k for the graphite (upper) and charcoal (lower) drawings indicate a significant imaging contrast difference between the two materials as a result of their different absorption properties^33, 38^. Nevertheless, the achieved S/N was adequate for the accurate identification of the underlying sketch in both cases, demonstrating thus the universality of the proposed photoacoustic approach. Finally, to characterize the canvas background effect due to the intense optical scattering by the fiber grid, we have used a similar pencil to darken homogeneously a bare canvas sample. The respective photoacoustic image shown in Fig. 2l reveals a well-defined pattern of imaging contrast loss throughout the scanned area, as a result of the negligible signal excitation in these regions.

**Figure 3:**
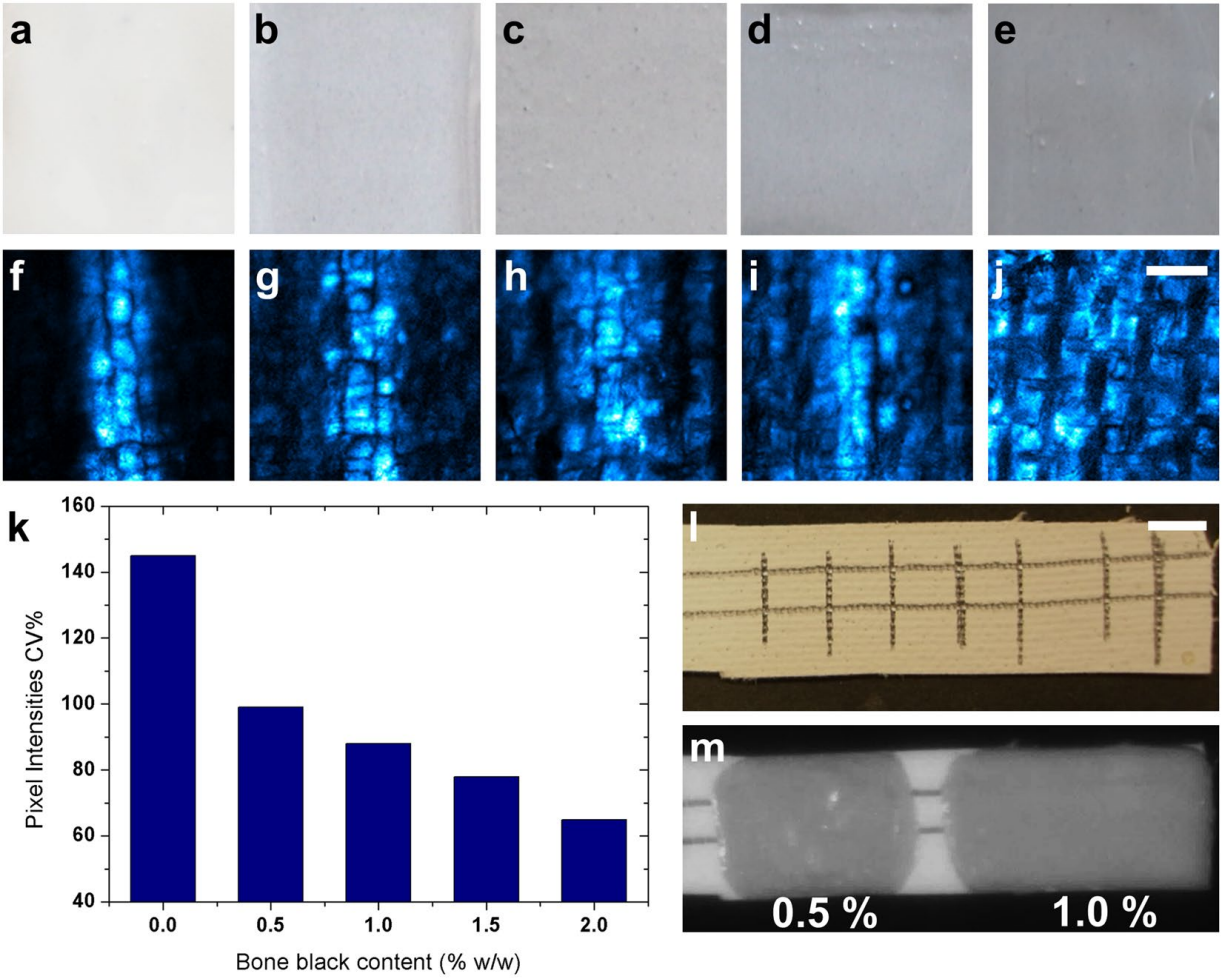
Sensitivity limits of photoacoustic imaging. (**a**–**e**) Brightfield images of canvas samples covered with oil paint layers containing zinc white and bone black pigments at gradually increasing concentration of the latter in the range of 0–2% at 0.5% increments. (**f**–**j**) Corresponding photoacoustic images revealing an underlying vertical line sketch presenting progressive contrast degradation for increasing bone black concentration. Scalebar: 1 mm. (**k**) Mean normalized standard deviation of pixel intensities (CV%) for images (**f**–**j**) versus bone black concentration, as a quantitative measure of contrast degradation. (**l**) Brightfield view of pencil lines sketched on canvas sample. (**m**) NIR optical image recorded at 1200 nm of the canvas sample shown in (**l**) coated with zinc white mixed with bone black paint at 0.5 and 1% by weight. Scalebar: 1 cm.

To further explore the sensitivity limits of photoacoustic imaging, test samples were examined in which pencil sketches were coated over with paint highly absorbing in the NIR. More specifically, a uniform, ~1 mm wide, line was drawn on a prepared canvas with a pencil, and was subsequently covered with zinc white oil paint mixed with small amounts of bone black, with the relative concentration of the latter being in the range of 0.5–2% by weight. By gradually adjusting the relative concentrations of these pigments in mixed paint layers, we actually controlled the optical behavior of the specimen, and rendered it from solely high scattering to both high scattering and absorbing, which is a case that corresponds to the toughest detection conditions. Despite its low content, bone black, a highly absorbing pigment, gave a significant grayish coloration to the paint as shown in Fig. 3a–e. The corresponding photoacoustic images (Fig. 3f–j) reveal a gradual reduction of imaging contrast as the bone black content increases, and this is a result of the background noise generated due to the strong absorption of the overlying paint layer. With this series of tests it was concluded that the underdrawing was not any more distinguishable when the bone black relative concentration reached 2%, since the photoacoustic signals induced by the pencil sketch and the black pigment were approximately of equal magnitude. A quantitative analysis of the imaging contrast was additionally performed by calculating the coefficient of variation (CV%) for the signal intensity recorded on each pixel in a given image:

2 $$CV \% =\frac{1}{\bar{I}}\sqrt{\frac{1}{XY}\sum _{i=0}^{X-1}\,\sum _{j=0}^{Y-1}{({I}_{ij}-\bar{I})}^{2}}$$

where *I*_*ij*_ is the i-th, j-th intensity element for a two-dimensional image with a size of X by Y pixels and $\bar{I}$ stands for the respective average pixel intensity. Viewed from another perspective, CV% represents the Root Mean Square (RMS) contrast of the image normalized with respect to the average pixel intensity. This method of contrast quantification permits a direct, unbiased comparison of images characterized by highly varying average intensity values, and is also insensitive to the spatial distribution of the signals. Figure 3k presents the CV% values for the respective five different paints with increasing bone black concentration, estimated for the corresponding photoacoustic images (Fig. 3f–j). These results indicate a drastic CV% decrease of almost 50% between the neat zinc white paint and the 0.5% bone black paint sample, followed by a gradual, approximately linear reduction on the order of 10% per 0.5% concentration increment. In parallel the capacity of conventional NIR imaging to detect underdrawings was tested with a similar paint sample bearing pencil lines (Fig. 3l) on prepared canvas painted over by two different paint layers of zinc white mixed with 0.5% and 1% bone black respectively. Optical imaging performed *en face* at 1200 nm (Fig. 3m) with a multi-spectral imaging camera (see Methods section) was found not capable of detecting the underlying pencil sketch in either case, as a result of the strong interaction of the incident light with the overlying pigments.

### Photoacoustic imaging of miniature paintings

**Figure 4:**
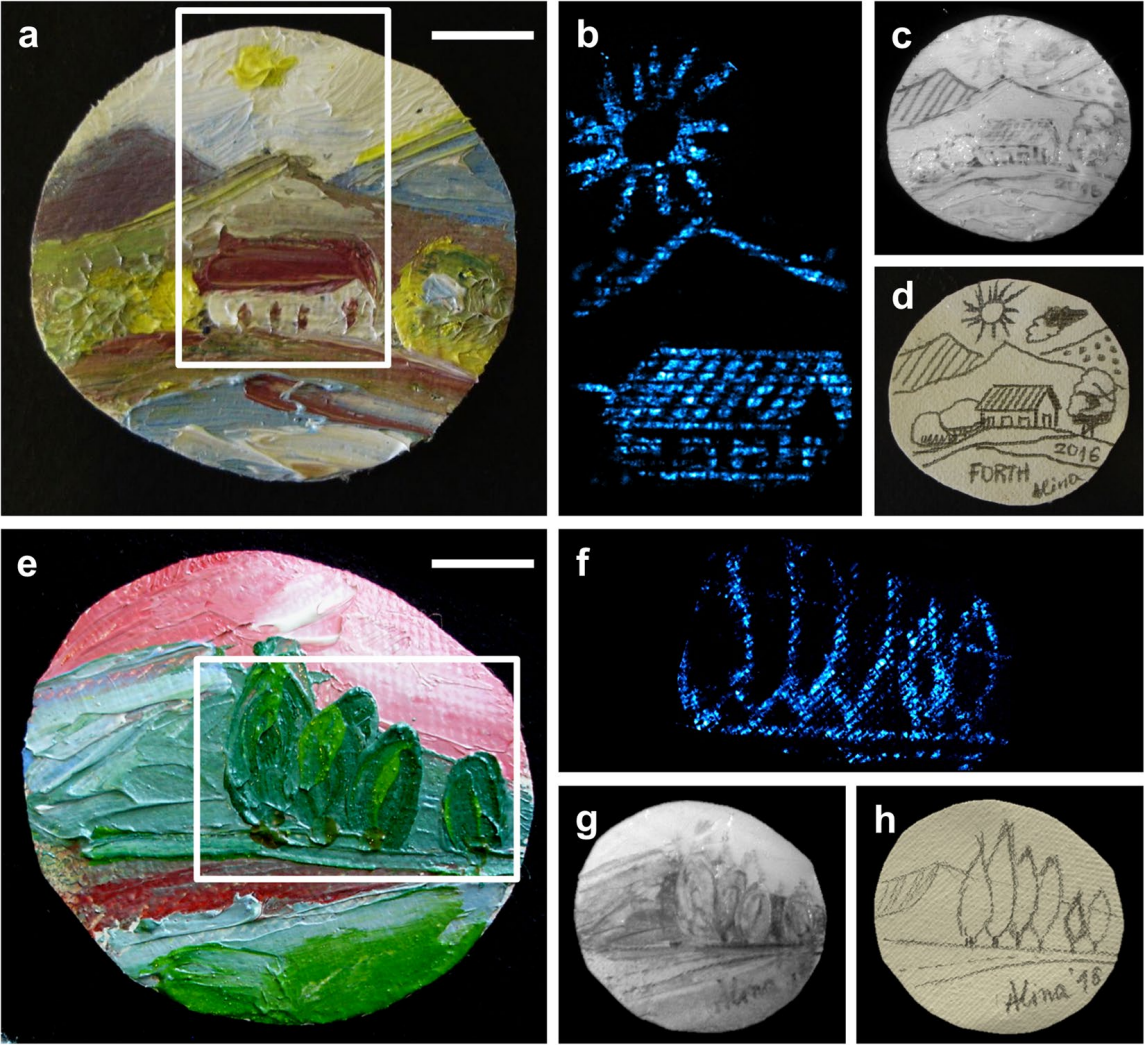
Photoacoustic detection of underdrawings in miniature paintings. (**a**) Brightfield view of miniature painting (rural landscape). (**b**) Photoacoustic image of the painting’s underlying sketch over a central region of 2.2 × 3.8 cm as indicated by the white box in (**a**). (**c**) NIR image of the underdrawing at 1200 nm. (**d**) Brightfield view of the original pencil sketch prior to overpainting. (**e**) Brightfield view of miniature painting (forest landscape). (**f**) Photoacoustic image of the underdrawing for the indicated region in (**e**) with lateral dimensions equal to 3.4 × 2.4 cm^2^. (**g**) NIR image of the painting at 1200 nm. (**h**) Original pencil sketch before overpainting. Both scalebars: 1 cm.

Having evaluated the performance and limitations of the photoacoustic modality on the imaging of samples covered by paint layers, we proceeded with the investigation of realistic, purposefully created miniature artworks painted using several mixed oil paints of diverse relative concentrations and thicknesses. The image of one of the miniature oil paintings portraying a rural landscape is shown in Fig. 4a. Pigments such as zinc white, ultramarine blue, cadmium red and chrome yellow were used for painting this miniature. A region close to the center of the painting with dimensions 2.2 × 3.8 cm^2^ (white box in Fig. 4a) was imaged by means of the photoacoustic microscopy system. It is noted that, with the given field of view of the present setup covering a circular area of diameter around 2 cm, the photoacoustic image shown (Fig. 4b) is a result of the vertical stitching of two sequential lateral scans. The image clearly reveals the underlying pencil drawing. For comparison, the painting was additionally imaged by the multispectral imaging camera at 1200 nm (Fig. 4c), whereas the initial sketch prior to the application of oil paints is shown in Fig. 4d.

To further verify the capabilities of the proposed methodology on the visualization of underdrawings, a second miniature painting was examined, portraying a forest landscape (Fig. 4e), and painted with a different pigment scheme. Following the preliminary pencil sketching, the artwork was overpainted using a combination of zinc white, chromium green, ultramarine blue, vermillion and azurite. As with the previous painting, a selected region of 3.4 × 2.4 cm^2^ (white box in Fig. 4e) was imaged as shown in Fig. 4f. The contrast of the revealed contours is similarly compared to the corresponding NIR image at 1200 nm (Fig. 4g), as well as, the original drawing before applying the paint (Fig. 4h). It is observed that in both miniature paintings, the photoacoustic images presented significantly higher contrast levels as to the accurate underdrawing mapping in comparison to the respective regions of the NIR images.

## Discussion

As noted in the introduction, diffusion of light has always been a limiting factor for optical diagnostic techniques and particularly in various types of light microscopy, given that scattering processes drastically affect the propagation of ballistic photons necessary for gaining useful imaging contrast when probing optically complex media. Paintings are highly scattering media and therefore strong diffusion dominates light transmission, severely restricting any in-depth investigation beyond the extent of a few hundreds of micrometers. On the contrary, photoacoustic imaging takes advantage of light scattering that enables excitation of a broad volume within the investigated medium, both axially and laterally, in a manner which is entirely independent of the directions of the interacting photons. Yet, high resolution mapping of absorbing features embedded within the medium can be obtained by deliberately limiting the region where photoacoustic signal is collected from, and this is achieved in the present setup by the use of a spherically focused ultrasound detector. It is indeed the focus of the ultrasonic transducer that determines the point imaged and in this manner, a point by point acquisition procedure directly generates the maximum photoacoustic amplitude image across the region of interest, effectively eliminating the need of using a back projection algorithm for image reconstruction.

Painted works of art are typically made on a prepared canvas, which is a thin fabric properly stretched, coated with a layer of white paint. This preparation layer consists of a white pigment, such as lead white, common in medieval and later times, replaced by titanium white from the early 20^th^ century, mixed with oil, glue and resin serving as binders^39^. These materials are generally characterized by a very high scattering coefficient, and as such are quite suitable media for photoacoustic imaging applications. Considering that the emphasis of the present work is placed on imaging underdrawings, the proposed approach has a significant advantage, as it relies on irradiating the painting from its reverse side, with the laser pulse energy being directly absorbed by the underdrawing. The photoacoustic waves generated as a result of light absorption are subsequently transmitted through the overlying paint towards the detector practically unobstructed, given the dramatically lower, more than three orders of magnitude, scattering coefficient values of ultrasound waves when compared to light^40^. This property of the photoacoustic signal explains why the contrast of the underdrawing images presented in this work does not appear to be strongly dependent on the paint layer thickness, in comparison to the results achieved through conventional NIR imaging, and obtained in the back-scattering mode following “front-face” illumination of the painting. This obviously involves double-passing of the light through the superficial paint layers resulting in strong signal attenuation and producing poor quality imaging of the underlying sketch. It is noteworthy that in cases of paint layers with high optical density, such as the ones shown in Fig. 3, photoacoustic imaging provided adequate contrast while imaging in the NIR did not succeed in resolving the overpainted sketch lines.

Despite the fact that the photoacoustic technique has provided high quality mapping of the hidden pencil sketches, the images appear to suffer from a small artifact due to the non-uniform scattering of the incident radiation from the canvas fibers. However, the reduction or even the loss of imaging contrast across a grid-like spatial pattern which is less than 0.5 mm thick, is expected to have negligible consequences on the recognition of an underdrawing in a real painting with typical size in the order of several tens of cm, especially if we take into account that a pencil’s tip diameter is usually around 1–2 mm or larger. In any case, this artifact could be practically eliminated by integrating a more sophisticated illumination scheme, including the simultaneous irradiation of the painting from both sides or from various angles in respect to the vertical axis, through the use of optical fibers. These proposed photoacoustic excitation configurations, would ensure a more homogeneous distribution of the incident photons into the painting as a result of their insensitivity to extremely strong scattering centers such as canvas fibers. Another approach towards this direction would be the post processing of the acquired images by employing specialized interpolation algorithms, which would fill the gaps in the reduced contrast regions, given the fact that the generated photoacoustic signals are not expected to vary significantly along distances which are comparable to the lateral resolution of the imaging system.

An important consideration related to the effectiveness of photoacoustic imaging in such an application is the selection of the appropriate excitation wavelength. In most of cases, underdrawings are generated using carbon-based pigments such as charcoal, graphite or carbon black. These pigments absorb highly in a broad spectral region ranging from the UV to NIR unlike most of the inorganic pigments used in painting, which feature significantly lower absorption in the NIR range compared to carbon. In this context, the widely available Nd:YAG laser emitting at 1064 nm appears ideal for this type of measurements given its selective absorption by the underdrawing material and its relatively high transmission through the overlying paint layers, as long as they do not contain significant amounts of carbon-based pigments. Nevertheless, it has to be mentioned that an analysis of the recorded waveforms was attempted for the bone black containing paint layers (the only ones to provide strong photoacoustic background), which, however, was not able to differentiate between the pencil sketch and the overlying paint due to the temporal overlap of the respective signals, implying that the relative distance of the absorbing components was smaller than the achieved axial resolution. A wider bandwidth detector could be employed in order to increase the axial resolving power, sacrificing though the maximum imaging depth, as a result of the intrinsically stronger attenuation of the higher frequency components during their propagation in a medium.

As regards the safety boundaries with respect to potential thermal effects of the laser irradiation on the artwork, the maximum local temperature rise was estimated for a single laser pulse incident on the substrate under typical thermal confinement conditions. It is noted that the prepared canvas constituents, fabric, glue, oil paint, are known to be materials of low thermal conductivity. Assuming validity of the thermal confinement approximation, the maximum limit of temperature rise was calculated to be ΔΤ = 12.6 K produced within the 10 ns time interval of the laser pulse. In reality, considering that not the entire amount of the laser pulse energy reaches the underdrawing, as part of it is scattered by the canvas substrate, and taking the thermal losses into account, ΔΤ can be even lower than the calculated maximum value. This ensures that during photoacoustic measurements temporary and localized temperature changes are maintained well within safety limits and far from levels that would induce any alterations in the painting materials^41^. Furthermore, due to the fact that most of the pigments contained in the oil paint layers are virtually transparent at 1064 nm, no photochemical alterations are expected to take place, even for relatively large energy fluences. Finally, it has to be mentioned that well-established non-destructive optical characterization techniques of paintings such as Raman, or laser-induced fluorescence spectroscopy, often use energy fluences and exposure times which are much higher than 0.085 mJ/cm^2^ and 10 ns respectively, without inducing any apparent damage^42, 43^. Therefore, all of this evidence indicates that the proposed photoacoustic imaging methodology is quite safe for the detailed and high contrast mapping of hidden underdrawings, with minimal disturbance on the examined artefact.

During the recent decades, cellulose ethers have been extensively used in art conservation as gelling agents^44^ for the surface cleaning of various artefacts such as frescos^45^, papers^46^, paintings^47^ and marbles^48^ due to their wide availability, low cost and safety both concerning conservators and the artworks themselves. Detailed studies employing radio-isotopic assays have additionally determined the critical solution parameters such as pH, concentration, viscosity etc. as to the minimization of the residual content on the artefact, following the end of the conservation process^49^. In the present study, a 3% w/v CMC gel layer has been used as a highly compatible immersion medium for the effective propagation of the generated photoacoustic signals to the ultrasonic detector. The CMC gel was in general easy to remove from the surface of the paintings studied at the end of each imaging session, without any apparent superficial alteration as verified following examination with an optical microscope. Obviously other advanced gel-type materials^50–53^ compatible with painted surfaces could be considered as appropriate for photoacoustic measurements. In the case of sensitive paint surfaces that could not tolerate contact with any gel material, the option of immersion medium-free photoacoustic imaging can be considered in the context of an upgraded version of the current setup incorporating a fully non-contact interferometric ultrasonic detector^54, 55^. Such an experimental implementation would further highlight the broad potential of photoacoustic imaging in artwork diagnostics as demonstrated by the current proof of concept study.

In conclusion, the development of a novel non-invasive imaging methodology based on the photoacoustic effect is demonstrated in the context of painting investigations with emphasis on the delineation of underdrawing and original sketch lines. Photoacoustic imaging offers increased detection sensitivity, excellent contrast levels and high spatial resolution as to the state of the art techniques. In addition to these features, the simplicity of the implementation and the relatively low cost of the experimental setup, render the proposed method appropriate for a broad spectrum of applications regarding art objects diagnostics. As a future work, we are aiming to develop a compact multi-wavelength photoacoustic imaging setup, which will be optimized exclusively for the analysis of various sized real artworks, in terms of geometrical, optical and acoustic parameters configuration. We anticipate that such a dedicated system will further attract the interest of the cultural heritage science community, paving the way for more relative contributions in this new research field.

## Methods

### Experimental setup

The photoacoustic microscopy imaging system employs a variable repetition rate diode pumped Nd:YAG laser emitting at 1064 nm (QIR-1064-200-S, CrystaLaser LC, Reno, NV, USA; pulse energy: 29.4 µJ, pulse duration: 10 ns, repetition rate: 6.78 kHz, M^2^ value: ~1.2). The beam is expanded by a factor of six using a telescope so as to fill the back aperture of the objective lens and subsequently attenuated through a set of neutral density filters that permit precise control of the energy incident on the sample. A 45° mirror reflects the infrared radiation into a modified inverted optical microscope (Labovert, Leitz, Wetzlar, Germany) which serves as a platform for the photoacoustic modality. A low numerical aperture (NA) objective lens (Achromat 8X, LOMO, St. Petersburg, Russia; air immersion, NA: 0.2) is used that ensures proper focusing of the beam on the back surface of the canvas. The average power on the sample was measured to be 7.7 mW, corresponding to a pulse energy of 1.14 μJ. The sample is placed into a custom-designed holder with a round window at its bottom, 2.5 cm in diameter, covered by a standard BK7 170 μm thick, microscope coverslip. The optical window is tightly sealed around by silicone gel to prevent leakage of the overlying immersion medium and provide robustness during the measurements. A high resolution motorized XY stage (8MTF-75LS05, Standa, Vilnius, Lithuania) raster scans the sample holder over the beam focus, whereas the respective vertical positioning is achieved through the manual Z-adjustment controls of the microscope. A spherically focused piezoelectric ultrasonic transducer (V373-SU, Olympus, Tokyo, Japan; central frequency 20 MHz; effective bandwidth: 13–33 MHz, focal distance: 32 mm, NA: 0.1) is immersed into the CMC gel covering the sample and is positioned in a confocal and coaxial configuration with respect to the illumination. The detected photoacoustic signals are further amplified by a low noise, high gain RF amplifier (AU-1291, Miteq, NY, USA; gain: 63 dB) and recorded through a 14-bit digitizer (PCIe-9852, ADLINK, Taipei, Taiwan; sampling rate: 200 MS/s; bandwidth: 90 MHz). Control and synchronization of the microscope’s devices are implemented in MATLAB programming environment, whereas data processing is performed using both MATLAB and ImageJ. Each pixel brightness value of the final 2D images corresponds to the maximum amplitude projection of sixteen averaged waveforms which are recorded for S/N enhancement. Due to the intense optical scattering, the lateral resolving degree is ultimately determined by the acoustic focus of the transducer, which yields a diffraction limited resolution on the order of 525 μm. Finally, the total time required for a continuous raster scanning of a sample at 300 by 300 pixels resolution is around 25 minutes.

### Preparation of canvas samples

Several samples have been prepared for this work, aiming to replicate real easel paintings in a small scale. More specifically, various geometric patterns and drawings were produced on prepared canvas (coated with a thin layer of titanium white paint, TiO_2_), using two different types of drawing material, a graphite pencil (CASTELL 9000 3B, Faber-Castell, Stein, Germany) and a charcoal stick (Artist’s Willow Charcoal - Medium, Winsor & Newton, London, UK). The pencil and charcoal drawings, representing the sketch layer of the painting, were covered with layers of various oil paints which exceeded 0.5 mm in thickness. All investigated paints were prepared by mixing the corresponding pigment in powder form with linseed oil used as binding medium (Bleached C1104, Sthebenignen, Drieberger, Holland) and are listed in the following table (Table 1).

**Table 1:** Pigments used for the preparation of paint layers.

Pigment	Chemical composition	Supplier
Zinc white	ZnO	Kremer 46300
Bone black	C + Ca_3_ (PO_4_)_2_	Kremer 4710
Cadmium red (No. 3, dark)	CdSe_x_S_1−x_	Kremer 2114
Vermillion	HgS	Sigma Aldrich, FLUKA
Chrome yellow	PbCrO_4_	Kremer 4370
Chromium green	Cr_2_O_3_	Kremer 44200
Ultramarine blue	Na_7_Al_6_Si_6_O_24_S_3_	Kremer 4503
Azurite	Cu(OH)_2_·CuCO_3_	MOSINTER GROUP LIMITED, GAS 12069-69-1 (China)

### CMC gel preparation

The immersion medium was prepared by adding solid Carboxymethyl cellulose (CMC) sodium salt in deionized water at 3% w/v concentration. The mixture is vigorously stirred at room temperature for approximately 30 minutes, until the formation of a colorless gel. The gel is further sonicated for 45 minutes in order to remove any bubbles formed during stirring.

### Spectral measurements of oil pigments

The diffuse reflectance spectra of powder pigments, in the form of thick (3 mm) pellets, were measured on a UV-Vis-NIR spectrophotometer equipped with an integrating sphere (Lambda 950, PerkinElmer, Waltham, MA, USA) in the range of 350–1200 nm. Prior to the actual spectral measurements, the spectrometer was calibrated using a highly reflective (>99%) Spectralon target (Labsphere, North Sutton, NH, USA) for defining the 100% on the reflectance scale. The absorption spectrum, A(λ) of each pigment is directly calculated through the corresponding reflectance spectrum, R(λ), as A(λ) = 1 − R(λ), assuming negligible sample transmittance (which is true considering the opacity of the pellets).

### Multispectral imaging system

Images in the NIR were recorded using a custom multispectral imaging system^56^ dedicated for paintings diagnostics. The system employs a high resolution CMOS camera (UI-5480CP, IDS, Obersulm, Germany; 4.92 MP), coupled with an IR transparent objective lens (Macro Lens 25 mm F 1.3, Electrophysics Corp., West Fairfield, NJ, USA), whereas the respective illumination is achieved using two broad band emission lamps (Halostar Starlite, OSRAM, Munich, Germany; 50 W, 12 V). For underdrawing detection, images were collected in the NIR, by positioning in front of the camera sensor a bandpass filter with central wavelength at 1200 nm and 25 nm bandwidth (1200BP25, Omega Optical, Brattleboro, VT, USA).
